# An ecological momentary assessment study of age effects on perceptive and non-perceptive clinical high-risk symptoms of psychosis

**DOI:** 10.1007/s00787-022-02003-9

**Published:** 2022-05-18

**Authors:** C. Michel, S. Lerch, J. R. Büetiger, R. Flückiger, M. Cavelti, J. Koenig, M. Kaess, J. Kindler

**Affiliations:** 1 University Hospital of Child and Adolescent Psychiatry and Psychotherapy, University of Bern, Bern, Switzerland; 2 Department of Child and Adolescent Psychiatry, Psychosomatics and Psychotherapy, University of Cologne, Faculty of Medicine and University Hospital Cologne, Cologne, Germany; 3 Department of Child and Adolescent Psychiatry, Centre for Psychosocial Medicine, University of Heidelberg, Heidelberg, Germany

**Keywords:** EMA, Experience sampling method, Basic symptoms, Psychosis risk, Perceptive symptoms

## Abstract

Among individuals with clinical high risk for psychosis (CHR), perceptive symptoms are more frequent but have less clinical significance in children/adolescents compared to adults. However, findings are based on clinical interviews relying on patient’s recall capacity. Ecological momentary assessment (EMA) can be used to explore experiences in real-time in the subject’s daily life. The aim of this study was to assess frequency and stability of (perceptive and non-perceptive) CHR symptoms and to explore potential age effects. EMA was used in a sample of an early detection for psychosis service in Bern, Switzerland (*N* = 66; 11–36 years). CHR symptoms were recorded in random time intervals for seven days: eight assessments per day per subject, minimum time between prompts set at 25 min. CHR symptoms were additionally assessed with semi-structured interviews including the ‘Structured Interview for Psychosis-Risk Syndromes’ and the ‘Schizophrenia Proneness Instruments’. Mixed-effects linear regression analysis on the frequency of CHR symptoms revealed a significant effect of age group, and the interaction CHR symptoms *x* age group for both perceptive and non-perceptive symptoms. Further, regarding stability of CHR symptoms, there was a significant effect of the interaction CHR symptoms *x* age group for perceptive symptoms only. Based on EMA, perceptive CHR symptoms were more frequently reported but less stable in children/adolescents compared with adults. Together with previous findings, our finding of higher instability/variability of perceptive symptoms in younger persons might suggest that with advancing age and more stability of CHR symptoms, clinical relevance (reduced psychosocial functioning) may increase.

**Supplementary Information:**

The online version contains supplementary material available at 10.1007/s00787-022-02003-9.

## Introduction

Everyday patient behaviour and the course of their symptomatology are of interest to health practitioners. Especially in clinical psychology and psychiatry, there is an increased awareness that psychological models of psychopathology are essentially dynamic over time, their functional impairments are expressed in real-world settings, and multiple assessments are required to understand their course [1, 2]. Ecological momentary assessment (EMA), also known as the experience sampling method, involves repeated sampling of subjects' current behaviours and experiences in real time, in subjects' natural environments by shedding light on the dynamics of behaviour in everyday life [2]. Typically, participants are provided with a mobile device (e.g., a smartphone) that is programmed to signal them multiple times per day over a series of days or weeks to answer brief, specific questions as they go about their daily life [3]. EMA aims to minimize recall bias, maximize ecological validity, and allows studying micro processes (i.e., how behaviour and experience vary over time and across changing contexts) that influence behaviour in real-world contexts [2, 3]. A wide range of psychiatric disorders have been explored using EMA in the last two decades including affective disorders, anxiety disorders, borderline personality disorder and psychotic disorders [1, 4, 5]. The assessment of psychotic experiences with EMA is particularly useful because there is a powerful rationale for investigating these experiences—including their frequency and stability—in the context in which they actually occur [4, 6]. Positive symptoms of psychosis have been explored extensively with EMA [3, 6, 7]. Recent studies not only used EMA to explore real-life experiences in individuals with first-episode psychosis, but also included individuals with a clinical high-risk (CHR) state of psychosis [8, 9] and found a correspondence between EMA and the used clinical interview [9].

Psychotic disorders are severe mental disorders with often chronic course that incur high costs and burden to both society and affected patients [10–13]. Since the 1980s, multiple retrospective studies reported an association of a negative outcome of first-episode psychosis with a longer duration of untreated—or rather, inadequately treated—first-episode psychosis, as well as with untreated illness, i.e., the untreated duration of both the initial prodrome and first-episode psychosis [14–17]. The majority of first-episode psychotic disorders are preceded by a prodromal phase in which a multitude of CHR symptoms, other mental health problems and psychosocial deficits occur, and during which help may be sought [18, 19]. This phase offers a unique point of intervention for indicated prevention that aims to reduce CHR symptoms and the associated distress, thereby postponing or preventing entirely the transition to frank (fully manifest) psychosis [20]. Two major sets of CHR criteria are used for the assessment of this state: (1) ultra-high risk (UHR) criteria, i.e., attenuated (APS) or brief (limited) intermittent psychotic symptoms (B(L)IPS) and genetic risk and functional decline (GRFD); and (2) basic symptom (BS) criteria, i.e., cognitive disturbances (COGDIS) and cognitive-perceptive basic symptoms (COPER) [18, 19]. The two symptomatic UHR criteria APS and B(L)IPS only include positive symptoms of psychosis where some insight into their abnormal nature is still present [21]. Although negative (attenuated) psychotic symptoms are present in the psychosis prodrome, they are not yet included in criteria to define a CHR state [22]. BSs are self-experienced subclinical disturbances in mental processes, which are instantaneously perceived with full insight into their abnormal nature as deviations from one's normal mental processes—that is to say, the individual immediately recognises that the experiences are not part of their normal mental experiences and that they may be a split from reality, such as a delusion (i.e., not having 100% conviction of its reality). As such, BS are clearly different from the content-related, externalized and/or observable persistent positive features or symptoms described in APS/B(L)IPS [23]. A subgroup of 14 cognitive and perceptive BS forms COPER and COGDIS and they can usually be assessed from age 8 onwards [24] (see eTable 1).

Recent findings suggest that the clinical significance of perceptive (i.e., hallucinations, perceptual disturbances) and non-perceptive (i.e., (attenuated) delusional ideas, cognitive basic symptoms) CHR symptoms (including BS and UHR) depend on age, with higher frequency, but less clinical significance (lesser association with functional deficits and the presence of mental disorders) of perceptive symptoms in younger persons [25, 26]. Thereby, perceptive symptoms included at least one visual or acoustic perceptive disturbance or (attenuated) hallucination; non-perceptive (cognitive) symptoms included thought interference, blockages, pressure, and perseveration, disturbances of receptive and expressive speech, of abstract thinking, or of discriminating between ideas and perceptions, captivation of attention by details of the visual field, inability to divide attention, unstable ideas of reference, derealisation, (attenuated) delusional ideas, and (attenuated) disorganized communication [25, 26]. This age-dependency was found in the general population [25–27] as well as in clinical samples [27, 28]. These findings suggest a possible relationship between BSs and brain maturation (i.e., neurobiological development), and between APS and the maturation of cognitive abilities [26, 29]. Nevertheless, despite these findings on age differences, it might be difficult for clinicians to differentiate if a perceptive symptom is a sign for a psychotic development or if it is primarily associated with another diagnostic entity (e.g., bipolar disorder, borderline personality disorder; [30]). Cognitive models on perceptual symptoms point to a shared impairment in inhibitory functions, emotional problems, and top-down mechanisms across different population groups [30].

Longitudinal studies have shown a fluctuation of CHR symptoms over months and years [31] and even those not converting to psychosis often still report distressing CHR symptoms/criteria with patterns changing over time [32, 33]. These results suggest that CHR symptoms fluctuate over longer time periods, yet it is unclear if these fluctuations are also present over shorter time periods (e.g., within one week). Using EMA is a promising option to gain more insight into the phenomenology and the course of perceptive and non-perceptive (i.e., (attenuated) delusional ideas, cognitive basic symptoms) CHR symptoms. EMA is a technology used to track fluctuations in experiences and prompt behavioural responses within the context of a person's daily life and preliminary support for the clinical utility of EMA in the treatment of psychotic disorders has been found [4]. Therefore, using EMA can not only help to assess possible symptom fluctuations, but from a clinical perspective, it might also provide important information to adapt psychotherapeutic interventions accordingly.

Thus far, EMA has mainly been used in the CHR status to explore associations between (attenuated) psychotic symptoms and stress, affective symptoms and trauma [7, 8, 34] with BSs being largely ignored in EMA research, neglecting an important aspect of the CHR state. However, BSs, by definition are self-experienced disturbances especially of perception and cognitive processes [24] and therefore, qualify to be assessed by EMA. Assessing BSs with EMA would allow a better understanding of the nature and course (i.e., stability and fluctuations) of these symptoms in the daily life of patients. Thus, the aim of this study was to utilise EMA in a clinical sample to not only assess (attenuated) psychotic symptoms, but also BSs, and to specifically explore any differences between perceptive and non-perceptive (i.e., (attenuated) delusional ideas, cognitive basic symptoms) CHR symptoms with a focus on the influence of age.

## Methods

### Sample

The study sample (*N* = 66) was recruited at the ‘Bern Early Recognition and Intervention Centre’ (FETZ Bern, www.upd.ch/fetz; [35]). The FETZ Bern is the only psychosis-risk detection centre in the Canton of Bern with a catchment area of approximately 1.5 million inhabitants; the centre screens ~ 80 patients/year (age 8–40 years) for CHR symptoms according to state-of-the-art guidelines [19, 20]. Apart from accepting patients who enrol on their own initiative, patients with various psychiatric symptoms are admitted to the FETZ Bern by physicians and psychosocial institutions whenever there is clinical suspicion of early psychotic development. The FETZ Bern targets help-seeking persons with putative psychotic symptoms or CHR symptoms between 8 and 40 years of age. Exclusion criteria are (1) past clinical diagnosis of any psychotic disorder according to DSM and ICD, (2) diagnosis of delirium, dementia, amnestic or other neurological disorders, and (3) general medical conditions affecting the central nervous system. Age is an important factor in the FETZ Bern with about half of the population being 18 years or older and half of the sample being younger than 18 years with the main age of the younger ones being between 13 and 17 years (44.5% of the total population) [35]. In the FETZ Bern around 39% of the assessed persons meet CHR, 21% already meet criteria for a past or present psychotic disorder and 40% have no CHR or psychosis, but another psychiatric diagnosis (39.4%). As in other samples in the FETZ Bern the majority of CHR patients have a comorbid current axis-I disorder, mainly an affective or anxiety disorder (55.8%) [35, 36]. The clinical basic assessment includes a psychopathological evaluation, a cognitive test battery, cerebral magnetic resonance imaging, and a routine blood screening [35]. The present study sample consisted of consecutive attendees assessed from January 2019 to October 2020.

Patients agreed to the anonymized scientific use of their data and further gave consent to additionally participate in a study on ‘Exploratory behavioural and biological investigation of psychosis risk symptoms in children, adolescents and adults’ where the use of EMA was included. We received approval for all procedures from the ethics committee of the Canton of Bern. All participants gave informed consent and, in the case of minors, parental informed consent with the child's assent was provided. All procedures contributing to this work comply with the ethical standards of the relevant national and institutional committees on human experimentation and with the Helsinki Declaration. The human research ethics committee of the Canton Bern approved the study (ID PB_2016-01,991, ID 2018–00,951).

### Assessment

#### Interview-based assessment of CHR symptoms

CHR symptoms and criteria (see eTable 1) were assessed using semi-structured interviews with good interrater reliability [24, 37, 38]. These interviews comprised the following:

1. the Structured Interview for Psychosis-Risk Syndromes (SIPS) [37] for UHR symptoms and criteria, i.e., APS and the APS criterion, psychotic symptoms, and the B(L)IPS criterion;
2. Schizophrenia Proneness Instrument, both the Adult version (SPI-A) [38] and the version for Children and Adolescents (SPI-CY) [24], for predictive basic symptoms and related criteria, i.e., COPER and COGDIS [39].

For the present analyses, CHR symptoms were defined by the presence of any one APS, B(L)IPS, and/or basic symptom, irrespective of the onset/worsening and/or frequency requirements of related CHR criteria. CHR symptoms were only rated if the phenomenon in question was not fully and better explained by another non-psychotic disorder or psychotropic drug use [37, 39]. Specifically, the detailed questions in the Appendix B of the SIPS [37] which allow for yes/no answers were used to rate if a single symptom (e.g., visual hallucinations) was present or not. The SPI-A/ SPI-CY [24, 38] rank basic symptoms on a severity scale according to the maximum frequency of their occurrence within the past 3 months ranging from 0 (absent = basic symptom has not occurred in the past 3 months) to 6 (extreme = basic symptom has occurred daily over sometime within the past 3 months). Symptoms may also be rated as 7 (basic symptom has always been present in same severity; trait), 8 (basic symptom is definitely present, but its frequency of occurrence is unknown) and 9 (the presence of basic symptom can neither be unambiguously ruled in nor out). For the purpose of this study, basic symptoms were recoded according to their presence into binary items for better correspondence with the binary SIPS items: 1 (presence) was assigned to scores between 1 and 8 (i.e. all scores that clearly indicate the presence of the basic symptoms) and 0 (absence) was assigned to scores of 0 and 9 (i.e., scores clearly indicating the absence of the basic symptoms or only its ambiguous presence).

#### Ecological momentary assessment

After the third diagnostics appointment (parallel to continued diagnostic assessment), patients who agreed to participate in the study received a smartphone with the movisensXS experience sampling application (Movisens GmbH, Karlsruhe, Germany). MovisensXS enables the programming of smartphones to function as electronic diaries. All patients received extensive instructions on the use of the smartphone and corresponding application. EMA assessments commenced the same day patients received the smartphone and carried the e-diary on seven consecutive days. The movisensXS application randomly emitted an acoustic prompting signal with a total number of eight assessments per day, per subject and a minimum time between prompts of 25 min. Prompting signals started at 8am and finished regularly at 10 pm. To maximise compliance and increase the probability of obtaining all assessments, patients were enabled to postpone each alarm once for 5, 10 or 15 min. Each completed response was automatically time-stamped by the application. Data were assessed, uploaded and stored pseudonymized on both devices and movisensXS servers. Upon completion of the seven days EMA period, patients returned the smartphones, were debriefed and financially compensated, receiving 10 CHF for taking part in the EMA.

At each prompt, patients were asked to rate 14 items to assess the basic symptoms relevant for COPER and COGDIS. Further, they were asked seven items to assess (attenuated) psychotic symptoms. The items were selected based on the questions provided in the SPI-A/SPI-CY [24, 38] and the Appendix B of the SIPS [37]. We were further guided by previous studies using EMA in psychosis research [34]. The wording of items was shortened so that the maximum time for filling in the EMA was no more than five minutes. Each item was rated on a visual analogue scale (VAS) ranging from 0 to 100 (‘not at all’ to ‘all the time’), where a higher score corresponds to a higher severity of the respective symptom (i.e., higher frequency) (eTable 2).

### Data pre-processing of the EMA variables

We calculated the mean value of the 21 CHR symptoms assessed with the EMA for each administration. For the current analyses we further distinguished perceptive and non-perceptive (i.e., (attenuated) delusional ideas, cognitive basic symptoms) CHR symptoms. Perceptive symptoms were BS13, 14, APS 1, 2, and 5 and non-perceptive (i.e., (attenuated) delusional ideas, cognitive basic symptoms) symptoms were BS1-BS12, APS 3, 4, 6, and 7 (for more details see eTable 2). Dynamic processes such as the fluctuation of symptom severity/frequency over time are best quantified using indices that take into account the temporal dependency of repeated measures [40, 41]. Calculating squared successive differences (SSDs) has shown adequacy in quantifying instability in previous studies [41]. First, SSDs were calculated, i.e., differences of consecutive assessments of the CHR symptoms were determined and squared for all participants. We calculated SSDs for intervals ≤ 220 min between assessments. To account for positively skewed distributions of the SSDs we extracted the square root. Finally, the mean square rooted SSD (RMSSD) per patient was determined by calculating an average over all SSDs of the respective outcome variable of each participant to quantify instability of symptoms.

Before continuing the next steps in the analyses, the mean and RMSSD scores were z-transformed in order to make them comparable to each other.

### Statistical analyses

Descriptive information is presented in the form of means, standard deviations, median, range and percentages.

Mixed-effects linear regression analyses were conducted with the standardized EMA ratings as dependent variables (i.e., mean or RMSSD of the 21 CHR symptoms). In the models the 21 EMA ratings were treated as repeated measures and therefore the ratings were grouped by subject, i.e., including a random intercept. A so-called CHR symptom indicator variable (values 1–21) allowed for the identification of each of the 21 EMA ratings per subject. In a confirmatory factor analyses (CFA) we checked that the items we assigned to the two symptom groups (perceptive vs. non-perceptive) loaded significantly on the two factors.

First, to get an estimate of the agreement between interview and EMA ratings, we examined if the interview score for CHR symptoms predicted the EMA rating for CHR symptoms. The mean EMA value for the CHR symptoms was used as the outcome, and the dichotomous interview variable (symptom present or absent in interview), the CHR symptom indicator variable, and their interaction were included as fixed factors.

Further, the effect of age group [children/adolescents (< 18 years; *n* = 39) vs. adults (≥ 18 years; *n* = 27)] on frequency (mean) as well as instability/variability (RMSSD) of CHR symptoms was examined. We used this method instead of various t-tests of age group difference for each individual symptom and pooled the symptoms in one model. For multivariate regression our study had too few data to estimate a covariance between each symptom, therefore we first checked that all symptoms rating correlated positively to ensure that using a random intercept is adequate.

We investigated the effect of age group for finer and finer granularity with respect to individual CHR symptom level. For this we defined four different (nested) models for mean and RMSSD as outcomes separately:

Model0: Nullmodel with no fixed effects (only random intercept).

Model1: Allow only overall age group difference (age effect constant for all 21 CHR symptoms).

Model2: Allow different age group differences for perceptive and non-perceptive CHR symptoms.

Model3: Allow different age group differences for each of the 21 CHR symptoms.

The models are nested and each model can be expressed using constraints with the fixed factors (1) age group, (2) CHR symptom indicator variable, and (3) their interaction.

To decide which of the four models was best, we compared them using Likelihood-ratio tests separately for the frequency and instability/variability (Model0_mean, Model1_mean, Model2_mean, Model3_mean; Model0_rmssd, Model1_rmssd, Model2_rmssd, Model3_rmssd). For the best fitting model, we calculated contrasts overall CHR symptoms and the finest symptom level according to the chosen model.

Further, we calculated four sensitivity analyses for (1) the effect of participants having not completed at least one-third of the beeps [1, 42], (2) the effect of manifest psychotic disorders, (3) a potential effect of the COVID-19 outbreak and global pandemic on the model, and (4) the effect of using age as a continuous variable in the model.

The significance level was set to *α* = 0.05. Statistical analyses were conducted using Stata/SE (Version 17; Stata Corporation LP, College Station, TX, US).

## Results

A total of 114 patients were eligible and 70 patients (61.4%) agreed to participate in the study. Due to technical problems, three patients were unable to complete the assessment (software update, smartphone turned off) and one patient returned the smartphone after two days of assessment as it was too stressful to answer the questions. Thus, these four patients were not included in the further analyses and the final sample for the analyses, comprised 66 subjects. The mean duration of questionnaire completion was 112.5 (SD = 105.8) s. Overall compliance with the EMA was good (72.9% completed prompts; adults: 78.1%; children/adolescents: 69.3%) and patients provided 3,862 reports in total. Four participants had less than one-third of the beeps (14.3%, 25.4%, 30.0%, 32.2%). Therefore, in sensitivity analyses we checked if excluding them would change the results. Nearly half of our sample (*n* = 34, 52.3%) started the EMA before the COVID-19 pandemic and 31 participants (47.7%) after the outbreak (based on a national wide lockdown that was announced in Switzerland from the 16th of March 2020 onwards). One participant started the EMA exactly around the time when the pandemic first started, therefore this participant was excluded from our analyses of a potential effect of COVID-19 on our results. Of the remaining patients (*n* = 66), 39 were children/adolescents (< 18 years) and 27 were adults (≥ 18 years). With regard to the diagnostic groups, 52 fulfilled a current CHR state, six patients fulfilled criteria for a psychotic disorder and seven patients neither fulfilled criteria for a CHR state nor were they psychotic, and one patient aborted the diagnostics. Sociodemographic and clinical data of the sample are displayed in Table 1. Results of the CFA with the factor loadings for both groups (perceptive vs. non-perceptive) are displayed in the Supplementary Material (eTable 3), with significant factor loadings for both groups.

**Table 1:** Sociodemographic and clinical characteristics of the EMA sample

	Total sample (*N* = 66)		Children/adolescents (*n* = 39)		Adults (*n* = 27)	
	*n*	%	*n*	%	*n*	%
Age (mean ± SD, median, range)	18.9 ± 4.5, 17.4, 11.5–36.4		16.1 ± 1.4, 16.3, 11.5–17.9		23.0 ± 4.3, 21.6, 18.3–36.4	
Gender (male)	35	53.0	18	46.1	17	63.0
Highest education						
Primary school or school for special needs (6 school years)	6	9.1	5	12.8	2	7.4
Secondary school (9–10 school years)	43	65.2	30	76.9	18	66.7
High school (12–13 school years)	10	15.2	3	7.7	7	25.3
SOFAS score (mean ± SD, median, range)	57.5 ± 13.4, 55.0, 31–88		57.2 ± 13.8, 60.0, 31–88		58.0 ± 13.1, 55.0, 31–85	
Any current axis-I disorder^a^	38	57.6	25	64.1	13	48.1
Any affective disorder	33	50.0	22	56.4	11	40.7
Any anxiety disorder	10	15.2	6	15.4	4	14.8
Any eating disorder	1	1.5	0	0	1	3.7
Any somatoform disorder	0	0	0	0	0	0
An obsessive–compulsive disorder	2	3.0	1	2.6	1	3.7
A posttraumatic stress disorder	2	3.0	0	0	2	7.4
Any current CHR or psychotic symptom^a^	58	87.9	34	87.2	24	88.9
Any current APS (score 3–5)	44	66.7	27	69.2	17	63.0
Any current BIPS or psychotic symptom (score 6)	11	16.7	6	15.4	5	18.5
Any current basic symptom	53	80.3	31	79.5	22	81.5

*SOFAS* social and occupational functioning assessment scale, *CHR* clinical high risk, *APS* attenuated psychotic symptoms, *BIPS* brief intermittent psychotic symptoms

^a^An overlap between CHR symptoms and any current axis-I disorder was found for *n* = 36 (54.5%)

### Agreement between interview and EMA ratings

The interview scores significantly predicted the EMA ratings with an overall model fit of $\chi_{(41)}^2$ = 97.20, *p* < 0.001. Scoring in the interview led to a 0.31 SD higher mean EMA rating ($\chi_{(1)}^2$ = 43.43, *p* < 0.001, *z* = 6.59, *p* < 0.001), meaning that there was small to medium agreement between interview ratings and EMA ratings across all CHR symptoms.

### Evaluation of the different models for frequency (Model1_mean-Model3_mean)

The Pearson correlation coefficient between mean EMA ratings varied between *r* = 0.23–0.96, justifying our model definition for using a random intercept.

For frequency the Likelihood-ratio test showed that the model including the interaction with CHR symptom group (Model2_mean: AIC = 2715.5) fitted the data significantly better than only age group as predictor (Model1_mean: AIC = 2723.7) with $\chi_2^2$ = 12.23, *p* = 0.002. With ‘Model3_mean’ an increase of the granularity to the 21 individual CHR symptoms (Model3_mean: AIC = 2776.7) did not improve the model fit significantly ($\chi_{(38)}^2$ = 14.82, *p* = 1.000), therefore ‘Model2_mean’ was chosen as the best model for CHR frequency.

### Evaluation of the different models for instability/variability (Model1_rmssd-Model3_rmssd)

The Pearson correlation coefficient between RMSSD EMA ratings varied between *r* = 0.08–0.87, justifying our model definition for using a random intercept.

For instability the Likelihood-ratio showed that the model including the interaction with CHR symptom group (Model2_rmssd: AIC = 2988.2) fitted the data significantly better than only age group as predictor (Model1_rmssd: AIC = 3002.7) with $\chi_{(2)}^2$ = 18.54, *p* < 0.001. With ‘Model3-rmssd’ an increase of the granularity to the 21 individual CHR symptoms (Model3_rmssd: AIC = 3046.9) did not improve the model fit significantly ($\chi_{(38)}^2$ = 17.26, *p* = 0.999), therefore ‘Model2_rmssd’ was chosen as the best model for CHR instability/variability.

### Mixed-effects linear regressions for the frequency of CHR symptom group (Model2_mean)

The mixed-effects linear regression for ‘Model2_mean’ with age group (children/adolescents vs. adults), CHR symptom group (non-perceptive vs. perceptive symptoms), and their interaction term as predictors provided a significant overall model (see Table 2). We found an overall age group effect ($\chi_{(1)}^2$ = 8.27; *p* = 0.004) with adults having a lower mean frequency of CHR symptoms than children/adolescents. Due to standardization there was no significant overall CHR symptom group effect ($\chi_{(1)}^2$ = 0.00; *p* = 1.000), but the interaction CHR symptom group *x* age was significant ($\chi_{(1)}^2$ = 12.28; *p* < 0.001), indicating that children/adolescents had more frequent non-perceptive (cognitive) and perceptive symptoms compared with adults, with the group difference for the perceptive symptoms being bigger than for the non-perceptive symptoms (Table 2, eFigure 1).

**Table 2:** Mixed-effects linear regression for ‘Model2_mean’ with age group (children/adolescents vs. adults), CHR symptom group (non-perceptive vs. perceptive symptoms), and their interaction term as predictor

Overall model: $\chi_{(3)}^2$ = 20.44; *p* < 0.001***						
	Contrast	SE	*z*	*p*	95%CI; lower	95%CI; upper
Age group effect^a^	0.547	0.190	2.88	0.004**	0.174	0.920
CHR symptom group effect^b^	5.16e -10	0.037	0.00	1.000	− 0.073	0.073
Age group × CHR symptom group effect^a,b^	0.265	0.076	3.50	0.000***	0.117	0.413
Age group effect for perceptive symptoms^a^	0.749	0.199	3.77	0.000***	0.359	1.139
Age group effect for non-perceptive symptoms^a^	0.484	0.191	2.53	0.011**	0.109	0.859

All means and standard deviations were first converted to z-scores before the regression analyses were performed

^a^For the age group adults were coded as 0 and children/adolescents as 1, therefore a positive value means lower frequency for adults

^b^For the CHR symptom group perceptive symptoms were coded as 0 and non-perceptive symptoms as 1, therefore a positive value means lower frequency for perceptive symptoms

^*^Significant at *p* < 0.05, **significant at *p* < 0.01, ***significant at *p* < 0.001

### Mixed-effects linear regressions for the instability/variability of CHR symptom group (Model2_rmssd)

The mixed-effects linear regression for ‘Model2_rmssd’ with age group (children/adolescents vs. adults), CHR symptom group (non-perceptive vs. perceptive symptoms), and their interaction term as predictors provided a significant overall model (see Table 3). The overall age group effect ($\chi_{(1)}^2$ = 2.10; *p* = 0.148) as well as due to standardization the overall CHR symptom group effect ($\chi_{(1)}^2$ = 0.00; *p* = 1.000) were non-significant, but the interaction CHR symptom group *x* age group was significant ($\chi_{(1)}^2$ = 18.67; *p* < 0.001), indicating that children/adolescents reported more instable perceptive symptoms compared with adults, while no group difference was found for non-perceptive symptoms (Table 3, eFigure 1).

**Table 3:** Mixed-effects linear regression for ‘Model2_rmssd’ with age group (children/adolescents vs. adults), CHR symptom group (non-perceptive vs. perceptive symptoms), and their interaction term as predictor

Overall model: $\chi_{(3)}^2$ = 20.77; *p* < 0.001***						
	Contrast	SE	*z*	*p*	95%CI; lower	95%CI; upper
Age group effect^a^	0.269	0.186	1.45	0.148	− 0.095	0.633
CHR symptom group effect^b^	− 1.14e–09	0.042	0.00	1.000	− 0.083	0.083
Age group × CHR symptom group effect^a^	0.374	0.086	4.32	0.000***	0.204	0.543
Age group effect for perceptive symptoms^a^	0.554	0.197	2.81	0.005**	0.167	0.940
Age group effect for non-perceptive symptoms^a^	0.180	0.186	0.96	0.335	− 0.186	0.546

All instability indices derived from the mean square rooted successive differences (RMSSD) and their standard deviations were first converted to z-scores before the regression analyses were performed

^a^For the age group adults were coded as 0 and children/adolescents as 1, therefore a positive value means lower frequency for adults

^b^For the CHR symptom group perceptive symptoms were coded as 0 and non-perceptive symptoms as 1, therefore a positive value means lower frequency for perceptive symptoms

^*^Significant at *p* < 0.05

**Significant at *p* < 0.01

***Significant at *p* < 0.001

### Sensitivity analyses

**Fig. 1:**
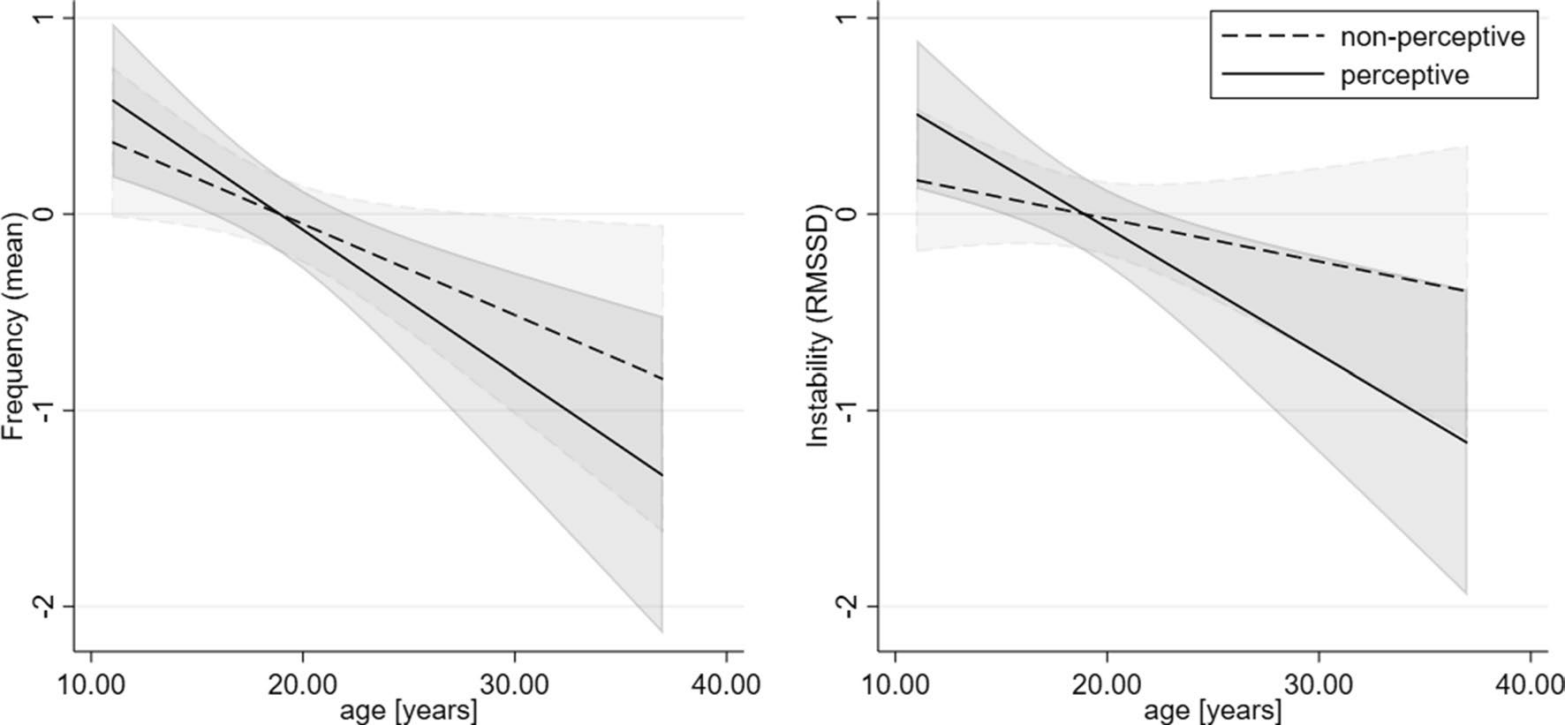
Effect of age as a continuous variable for both standardized outcomes (mean and RMSSD) on overall CHR symptoms and symptom group level (non-perceptive vs. perceptive) with 95% confidence interval

The four sensitivity analyses did not change the results of the model. (1) Excluding the four participants with not having completed at least one-third of the beeps did not change the results (see eTables 4, 5). (2) The results did not change when the model was calculated without the six patients who already had a manifest psychotic disorder (see eTables 5, 7). (3) Further, also considering the potential influence of the COVID-19 outbreak did not change the results of the model (see eTables 8, 9). (4) Including age as continuous variable still revealed lower frequency for both symptom groups with higher age ($\chi_{(1)}^2$ = 10.67; *p* = 0.001), with a significant effect of both perceptive and non-perceptive symptoms (eTable 10). Further, for both symptom groups stability increased with higher age ($\chi_{(1)}^2$ = 20.56; *p* < 0.001) with a significant effect for perceptive, but not for non-perceptive symptoms (eTable 11). The results of the continuous age variable are illustrated in Fig. 1.

## Discussion

This is the first study to utilise EMA in a clinical sample from an early detection of psychosis service to assess both (attenuated) psychotic symptoms and BSs, and to explore any age differences in CHR symptoms overall as well as in perceptive and non-perceptive (i.e., (attenuated) delusional ideas, cognitive basic symptoms) CHR symptoms separately. The two main findings of our current study are (1) that younger patients (children/adolescents) reported more frequent perceptive and non-perceptive CHR symptoms compared to older patients (adults) with larger group differences for perceptive than non-perceptive symptoms; and, (2) perceptive CHR symptoms in minor patients exhibited more variability (i.e., greater fluctuation of symptoms between EMA ratings) than in adult patients.

### Age effects on frequency of CHR symptoms

Findings of the higher frequency of perceptive (i.e., unusual perceptual experiences and attenuated hallucinations) symptoms, compared to non-perceptive (i.e., (attenuated) delusional ideas, cognitive basic symptoms) in children/adolescents compared to adults, is in line with previous research. Specifically, higher frequency of perceptive CHR symptoms in younger age [25, 27, 28, 43]. Further, previous findings regarding the clinical significance of perceptive CHR symptoms suggest that they also depend on age—with higher frequency, but less significance in younger persons. [25–29]. Children aged 7–8 years in the community have prevalence of 9% of APS, especially auditory hallucinations [44], that remit spontaneously in up to 75% [45]. Adolescents have even higher prevalence rates of up to 39% [46], which might gain significance when persistent to late adolescence [25]. Differential age effects of perceptual and non-perceptual CHR might follow normative brain maturation processes, in which they might occur as infrequent and temporary non-pathological disturbances. Their persistence or occurrence after conclusion of main brain maturation processes, however, might signify aberrant maturation or neurodegenerative processes [26, 29].

### Age effects on instability/variability of CHR symptoms

The finding that younger patients exhibit more variability in perceptive CHR symptoms is novel. Previous studies have not reported fluctuations of CHR symptoms over time. Nevertheless, it is in line with findings on daily fluctuations of auditory verbal hallucinations in patients with schizophrenia [47]. Our findings of perceptive CHR symptoms not only being more frequent but also more unstable/variable in younger persons, together with previously reported findings of lower clinical relevance in younger persons [25, 27, 28, 43], suggest that for older persons with more stability of perceptive CHR symptoms, the clinical relevance may increase. That is to say, if perceptive symptoms are less variable (i.e., more stable) in adult patients, this might signal more cause for concern, as symptom stability might indicate pathological brain processes (e.g., neuroinflammatory or neurodegenerative), rather than normative brain development with less clinical relevance (i.e., lesser association with functional deficits and presence of mental disorders) exhibited in younger persons. Further, for younger age groups, if symptoms have previously been unstable or variable, and now are more stable, then this also could be cause for concern. However, more research into other factors (e.g., content, frequency, loudness or beliefs about the perceptive symptoms) that might impact clinical distress should be studied together with EMA and related to objective measures, such as imaging-based tools in future research to understand more about the potential neurobiological changes associated with it.

### Clinical implications

The clinical evaluation of perceptive symptoms such as hallucinations in children and adolescents is a major challenge for physicians. Hallucinations may be associated with evolving psychosis but can alternatively occur as part of other psychopathologies (e.g., posttraumatic stress disorder, borderline personality disorder, autism spectrum disorder) or can occur in healthy individuals without any relation to a clinical disorder [30, 48–53]. Temporal dynamics, ability to distance from hallucinations, psychological distress, reduction in psychosocial functioning directly related to perceptions, and age at onset are important characteristics [53, 54] guiding clinical decisions with potentially serious consequences (e.g., introduce antipsychotic medication vs. start trauma-therapy vs. monitoring of symptoms). A recent review on hallucinations indicated an age of onset in late adolescence might be associated with the diagnosis of schizophrenia [53]. Authors concluded that focusing on other features may be particularly valuable in distinguishing schizophrenia from other disorders. Our data together with previous findings [25, 26, 28, 29] indicate that not only frequency but additionally stability of symptoms over time might be another important factor in the evaluation of perceptive symptoms, as higher stability of perceptive symptoms might signify higher clinical relevance. Therefore, systematically asking about onset of symptoms as well as assessing and monitoring the course of CHR symptoms not only over longer periods [32, 33], but also over shorter periods of time might be important for clinicians, in order to not only guide clinical decisions (i.e., diagnostics) but also to tailor specific therapeutic interventions that might be indicated if symptoms are stable over time. Further, EMA might also be used to make regular checks of the symptoms and track their evolution/change, to then adapt treatment over time, without conducting time-consuming and expensive clinical interviews every time. Clinicians not having the opportunity to use EMA with patients that present with frequent but variable symptoms could monitor the symptoms closely using the app Robin Z [55], the notes function of smartphones, diary cards as used in dialectical behavioural therapy, or offer regular calls to check whether symptoms have changed in their frequency and psychosocial functioning is affected.

Generally, in the early detection of psychosis there is still more research required to better stratify the different levels of risk (i.e., symptom manifestations or combinations) and provide individualization of treatment. Nevertheless, promising options are clinical staging models reflecting the dynamic nature of psychopathology, adapting the original CHR approach to encompass a broader range of inputs and outputs [56, 57]. In contributing to this, EMA might help to monitor symptoms and their dynamic changes more closely, to gather more information as to whether CHR symptoms are a transdiagnostic risk factor or dimension (i.e., suspiciousness/persecutory ideas; derealisation) or specific for psychotic disorders [58]. More specifically, stability/instability of symptoms might guide treatment in the future by providing a particular clinical indication for patient care. For example, it could be anticipated that an older individual with clinical presentation of higher symptom stability (with more clinical relevance such as higher levels of distress and poorer quality of life) might indicate a need for more prompt intervention, including higher levels of monitoring and psychosocial support to help manage distressing symptoms and other related outcomes.

### Strengths and limitations

#### Strengths

Our study has several strengths and limitations. A major strength is the use of EMA along with high-quality standard interviews of CHR symptoms. EMA provides significant innovative opportunities for in-vivo diagnostic assessment of state-dependent symptoms in everyday life, and can help to support clinical diagnoses and decision-making. Further, the assessment of symptom instability/variability and frequency—here in the domains of perceptive and non-perceptive CHR symptoms—may provide additional information on the severity and time-dependence of symptoms, thus allowing for relative evaluation and grading of symptoms.

#### Limitations

The cross-sectional design limits predictive ability for clinical outcomes. Further, EMA measures of CHR symptoms are based on subjective reports, however, we found a significant association between interview-based ratings and EMA ratings suggesting that there is agreement between interview ratings and EMA ratings. Another limitation of our study is that we only analysed between subject differences and we did not run any within subject analyses which would have a higher internal validity and offer a substantial boost in statistical power [59]. In future, analyses not only between subjects but also within subjects should be conducted.

### Future directions

The future of mobile technology in mental health care is growing substantially in the literature and might have a significant impact on clinical practice. A number of articles have described the various ways in which these technologies can be used in treating psychotic disorders [4] and assessing CHR states of psychoses [8]. Our finding of a higher frequency and variability of perceptive CHR in children and adolescents calls for their clinical re-appraisal in this age group in the early detection of psychosis, with the use of health-related applications or other mobile technologies. This is reflected in promising findings on auditory verbal hallucinations as part of early onset psychosis, whereby authors found feasibility and acceptability of a mobile application measuring and characterising these psychotic experiences. They concluded that using an application to assess symptoms might increase diagnostic resolution and could have therapeutic effects if symptoms correspond with similar fluctuations in cognitive control and experienced distress [60]. Information gained through applications could then be used to offer personalized and tailored psychotherapeutic interventions, e.g., for the treatment of psychosis risk symptoms also using ecological momentary interventions [4]. Further, future studies should also not only analyse between subject differences as we did with our approach, but also focus on within subject analyses to retain the power of EMA, which allows for analysis of repeated measures in more detail, and the dependence of symptoms on contextual variables. Additionally, within subject designs have a higher internal validity and they offer a substantial boost in statistical power [59].

## Conclusion

In “conclusion”, as the early detection of psychosis is increasingly applied to ever younger age groups, the need to re-evaluate the validity and clinical significance of current CHR criteria and symptoms in younger age groups should be addressed in future studies to improve understanding of what properties (such as age-at-onset, frequency and persistence) convey their clinical relevance at different developmental levels. In future, the combination of innovative technologies (e.g., apps) with objective measures, such as neuroimaging, should be given additional attention to gain further insight into the pathogenesis of psychosis and its early symptoms. Such studies have the potential to add to further development of useful targets for interventions and ultimately, improve clinical outcomes.

### Supplementary Information

Below is the link to the electronic supplementary material.

Supplementary file1 (DOCX 84 KB)

## Data Availability

The data that support the findings of this study are available on request from the corresponding author. The data are not publicly available due to privacy or ethical restrictions.
