# Supplementary material for: An ecological momentary assessment study of age effects on perceptive and non-perceptive clinical high-risk symptoms of psychosis
Source: Eur Child Adolesc Psychiatry. 2022 May 18;32(10):1841–52. doi: 10.1007/s00787-022-02003-9 (PMC9116495; doi:10.1007/s00787-022-02003-9)
Supplement: Supplementary file 1 — Supplementary file1 (DOCX 84 KB) [file 787_2022_2003_MOESM1_ESM.docx]

# Supplementary Material to:

## **An ecological momentary assessment study of age effects on perceptive and non-perceptive clinical high-risk symptoms of psychosis**

Michel C, Lerch S, Büetiger JR, Flückiger R, Cavelti M, Koenig J, Kaess M, Kindler J

**Content: 11 eTables, 1 eFigure**

- eTable 1: Clinical high-risk symptoms and criteria of first-episode psychosis
- eTable2: The 21 questions with regard to CHR symptoms asked in the ecological momentary assessment
- eTable 3. Factor loadings of the mean and RMSSD EMA scores on the two factors perceptive (P) and non-perceptive (N)
- eTable 4 and eTable 5: Results of the sensitivity analyses when excluding the four participants with less than one-third of beeps completed (n=62)
- eTable 6 and eTable 7: Results of the sensitivity analyses when excluding the six participants with already a manifest psychotic disorder (n=60)
- eTable8 and eTable 9: Results of the sensitivity analyses for the COVID-19 outbreak and global pandemic. Excluding one patient who used the EMA in the week when the lockdown in Switzerland was announced (n=65)
- eTable 10 and eTable 11: Results of the sensitivity analyses for age as continuous variable (n=66)
- eFigure1: Contrasts children/adolescents vs. adults for both standardized outcomes (mean and RMSSD) on overall CHR symptoms and symptom group level (non-perceptive vs. perceptive) with 95% confidence interval.

**eTable 1: Clinical high-risk symptoms and criteria of first-episode psychosis**

| **Ultra-high risk (UHR) criteria** according to the SIPS |
| --- |
| A. ‘Brief Intermittent Psychotic Symptoms’ (BIPS)  ⮊ At least any 1 of the following SIPS P-items scored 6 ‘severe and psychotic’   - P1 Unusual Thought Content / Delusional Ideas - P2 Suspiciousness / Persecutory Ideas - P3 Grandiose Ideas - P4 Perceptual Abnormalities / Hallucinations - P5 Disorganized Communication   ⮊ First appearance in the past three months  ⮊ Present for at least several minutes per day at a frequency of at least once per month but less than 7 days |
| B. ‘Attenuated Positive Symptoms’ (APS)  ⮊ At least any 1 of the following SIPS P-items scored 3 ‘moderate’ to 5 ‘severe but not psychotic’   - P1 Unusual Thought Content / Delusional Ideas - P2 Suspiciousness / Persecutory Ideas - P3 Grandiose Ideas - P4 Perceptual Abnormalities / Hallucinations - P5 Disorganized Communication   ⮊ First appearance within the past year or current rating one or more scale points higher compared to 12 months ago  ⮊ Symptoms have occurred at an average frequency of at least once per week in the past month |
| C. ‘Genetic Risk and Deterioration’ Syndrome  (1) Patient meets criteria for Schizotypal Personality Disorder according to SIPS  (2) Patient has 1^st^ degree relative with a psychotic disorder  (3) Patient has experienced >30% drop in global assessment of functioning (GAF) score over the last month compared to 12 months ago  ⮊ [1 and 3] or [2 and 3] or all are met. |
| **Basic symptom criteria** |
| Risk criterion ‘Cognitive-Perceptive Basic Symptoms’ (COPER)  ⮊ At least any 1 of the following basic symptoms with a SPI-A score of ≥3 within the last 3 months:   - Thought interference (BS3) - Thought blockages (BS4) - Thought pressure (BS5) - Thought perseveration (BS6) - Disturbance of receptive speech (BS7) - Unstable ideas of reference (BS10) - Decreased ability to discriminate between ideas and perception, fantasy and true memories (BS11) - Derealisation (BS12) - Visual perception disturbances (excluding hypersensitivity to light or blurred vision) (BS13) - Acoustic perception disturbances (excluding hypersensitivity to sounds) (BS14)   ⮊ First occurrence ≥12 months ago |

| High-risk criterion ‘Cognitive Disturbances’ (COGDIS)  ⮊ At least any 2 of the following basic symptoms with a SPI-A score of ≥3 within the last 3 months:   - Inability to divide attention (BS1) - Captivation of attention by details of the visual field (BS2) - Thought interference (BS3) - Thought blockages (BS4) - Thought pressure (BS5) - Disturbance of receptive speech (BS7) - Disturbance of expressive speech (BS8) - Disturbances of abstract thinking (BS9) - Unstable ideas of reference (BS10) |
| --- |

| **eTable 2: The 21 questions with regard to CHR symptoms asked in the ecological momentary assessment** | |
| --- | --- |
| Since the last rating…. | |
| BS1 | ... I found it difficult to divide my attention. |
| BS2 | ... my attention was caught by an unimportant detail from the environment. |
| BS3 | ... my concentration was disturbed by unimportant, off-topic thoughts. |
| BS4 | ... my thoughts were interrupted or blocked. |
| BS5 | ... I had a chaos of unrelated thoughts in my head and could not suppress new thoughts. |
| BS6 | ... petty thoughts or memories kept popping up and repeating themselves. |
| BS7 | ... I had problems understanding simple words or sentences. |
| BS8 | ... I felt I had to think longer to find the right words or phrases. |
| BS9 | ... I took everything too literally. |
| BS10 | ... I falsely referred to things in my environment. |
| BS11 | ... I was unsure whether I had actually experienced something or only imagined it. |
| BS12 | ... the world seemed completely different, somehow unreal, surreal and I felt like I was under a glass bell jar. |
| BS13 | ... my eyes were playing tricks on me and I saw familiar things in a different way. |
| BS14 | ... my ears were playing tricks on me and I heard familiar things in a different way. |
| APS1 | ... I have seen or perceived things that others have not seen. |
| APS2 | ... I have heard sounds/voices that others have not heard. |
| APS3 | ... I have felt suspicious or sceptical of others. |
| APS4 | ... I could not control my own ideas or thoughts. |
| APS5 | ... my thoughts were so intense that I could almost hear them. |
| APS6 | ... I had ideas that others found unusual or strange. |
| APS7 | ... I worried that something was wrong with my mind. |

*Note:* CHR: clinical high risk; BS: basic symptoms; APS: attenuated psychotic symptoms; Non-perceptive (cognitive) CHR symptoms: BS1-BS12, APS3, APS4, APS6, and APS7; Perceptive CHR symptoms (highlighted in grey): BS13, BS14, APS1, APS2, and APS5.

| **eTable 3.** Factor loadings of the mean and RMSSD EMA scores on the two factors perceptive (P) and non-perceptive (N) | | | | |
| --- | --- | --- | --- | --- |
| *MEAN* | *Factor N* | | *Factor P* | |
|  | Coefficient | p-value | Coefficient | p-value |
| Item 1(BS1) | 1.000 | 0.000*** |  |  |
| Item 2 (BS2) | 0.973 | 0.000*** |  |  |
| Item 3 (BS3) | 1.034 | 0.000*** |  |  |
| Item 4 (BS4) | 1.034 | 0.000*** |  |  |
| Item 5 (BS5) | 1.007 | 0.000*** |  |  |
| Item 6 (BS6) | 0.977 | 0.000*** |  |  |
| Item 7 (BS7) | 0.891 | 0.000*** |  |  |
| Item 8 (BS8) | 0.899 | 0.000*** |  |  |
| Item 9 (BS9) | 0.838 | 0.000*** |  |  |
| Item10 (BS10) | 0.878 | 0.000*** |  |  |
| Item 11 (BS11) | 0.800 | 0.000*** |  |  |
| Item 12 (BS12) | 0.510 | 0.000*** |  |  |
| Item 13 (BS13) |  |  | 1.000 | 0.000*** |
| Item 14 (BS14) |  |  | 1.046 | 0.000*** |
| Item 15 (APS1) |  |  | 0.952 | 0.000*** |
| Item 16 (APS2) |  |  | 0.933 | 0.000*** |
| Item 17 (APS3) | 0.930 |  |  |  |
| Item 18 (APS4) | 0.884 |  |  |  |
| Item 19 (APS5) |  |  | 0.767 | 0.000*** |
| Item 20 (APS6) | 0.910 |  |  |  |
| Item 21 (APS7) | 0.946 |  |  |  |
| *RMSSD* | *Factor N* | | *Factor P* | |
|  | Coefficient | p-value | Coefficient | p-value |
| Item 1(BS1) | 1.000 | 0.000*** |  |  |
| Item 2 (BS2) | 1.298 | 0.000*** |  |  |
| Item 3 (BS3) | 1.011 | 0.000*** |  |  |
| Item 4 (BS4) | 1.271 | 0.000*** |  |  |
| Item 5 (BS5) | 0.998 | 0.000*** |  |  |
| Item 6 (BS6) | 0.939 | 0.000*** |  |  |
| Item 7 (BS7) | 1.080 | 0.000*** |  |  |
| Item 8 (BS8) | 0.989 | 0.000*** |  |  |
| Item 9 (BS9) | 1.261 | 0.000*** |  |  |
| Item10 (BS10) | 1.281 | 0.000*** |  |  |
| Item 11 (BS11) | 1.073 | 0.000*** |  |  |
| Item 12 (BS12) | 0.755 | 0.000*** |  |  |
| Item 13 (BS13) |  |  | 1.000 | 0.000*** |
| Item 14 (BS14) |  |  | 1.031 | 0.000*** |
| Item 15 (APS1) |  |  | 0.921 | 0.000*** |
| Item 16 (APS2) |  |  | 0.942 | 0.000*** |
| Item 17 (APS3) | 1.147 | 0.000*** |  |  |
| Item 18 (APS4) | 1.279 | 0.000*** |  |  |
| Item 19 (APS5) |  |  | 0.774 | 0.000*** |
| Item 20 (APS6) | 1.053 | v |  |  |
| Item 21 (APS7) | 0.892 |  |  |  |

**Results of the sensitivity analyses when excluding the four participants with less than one-third of beeps completed (n=62).**

| **eTable 4**. Mixed-effects linear regression for ‘Model2_mean’ with age group (children/adolescents vs. adults), CHR symptom group (non-perceptive vs. perceptive symptoms), and their interaction term as predictor | | | | | | |
| --- | --- | --- | --- | --- | --- | --- |
|  | Contrast | SE | z | *p* | 95%CI; lower | 95%CI; upper |
| Age group effect^a^ | 0.633 | 0.195 | 3.254 | 0.001** | 0.252 | 1.015 |
| CHR symptom group effect^b^ | -0.023 | 0.038 | -0.610 | 0.542 | -0.097 | 0.051 |
| Age group x CHR symptom group effect^a,b^ | 0.272 | 0.077 | 3.554 | 0.001** | 0.117 | 0.413 |
| Age group effect  for perceptive symptoms^a^ | 0.749 | 0.199 | 3.767 | 0.000*** | 0.360 | 1.139 |
| Age group effect  for non-perceptive symptoms^a^ | 0.484 | 0.191 | 2.532 | 0.011* | 0.110 | 0.859 |

*Note:* All means and standard deviations were first converted to z-scores before the regression analyses were performed.

^a^For the age group adults were coded as 0 and children/adolescents as 1, therefore a positive value means lower frequency for adults.

^b^For the CHR symptom group perceptive symptoms were coded as 0 and non-perceptive symptoms as 1, therefore a positive value means lower frequency for perceptive symptoms.

*significant at p<0.05, **significant at p<0.01, ***significant at p<0.001

| **eTable 5**. Mixed-effects linear regression for ‘Model2_rmssd’ with age group (children/adolescents vs. adults), CHR symptom group (non-perceptive vs. perceptive symptoms), and their interaction term as predictor | | | | | | |
| --- | --- | --- | --- | --- | --- | --- |
|  | Contrast | SE | z | *p* | 95%CI; lower | 95%CI; upper |
| Age group effect^a^ | 0.314 | 0.188 | 1.671 | 0.095 | -0.054 | 0.682 |
| CHR symptom group effect^b^ | 0.009 | 0.043 | 0.203 | 0.839 | -0.076 | 0.093 |
| Age group x CHR symptom group effect^a^ | 0.371 | 0.087 | 4.248 | 0.000*** | 0.200 | 0.542 |
| Age group effect for perceptive symptoms^a^ | 0.596 | 0.199 | 2.993 | 0.003** | 0.206 | 0.987 |
| Age group effect for non-perceptive symptoms^a^ | 0.225 | 0.189 | 1.193 | 0.233 | -0.145 | 0.596 |

*Note:* All instability indices derived from the mean square rooted successive differences (RMSSD) and their standard deviations were first converted to z-scores before the regression analyses were performed.

^a^For the age group adults were coded as 0 and children/adolescents as 1, therefore a positive value means lower frequency for adults.

^b^For the CHR symptom group perceptive symptoms were coded as 0 and non-perceptive symptoms as 1, therefore a positive value means lower frequency for perceptive symptoms.

*significant at p<0.05, **significant at p<0.01, ***significant at p<0.001

**Results of the sensitivity analyses when excluding the six participants with already a manifest psychotic disorder (n=60).**

| **eTable 6**. Mixed-effects linear regression for ‘Model2_mean’ with age group (children/adolescents vs. adults), CHR symptom group (non-perceptive vs. perceptive symptoms), and their interaction term as predictor | | | | | | |
| --- | --- | --- | --- | --- | --- | --- |
|  | Contrast | SE | z | *p* | 95%CI; lower | 95%CI; upper |
| Age group effect^a^ | 0.477 | 0.189 | 2.519 | 0.012* | 0.106 | 0.848 |
| CHR symptom group effect^b^ | 0.003 | 0.038 | 0.080 | 0.936 | -0.072 | 0.078 |
| Age group x CHR symptom group effect^a,b^ | 0.230 | 0.077 | 2.975 | 0.003** | 0.079 | 0.382 |
| Age group effect  for perceptive symptoms^a^ | 0.652 | 0.198 | 3.289 | 0.001** | 0.264 | 1.041 |
| Age group effect  for non-perceptive symptoms^a^ | 0.422 | 0.190 | 2.219 | 0.027* | 0.049 | 0.795 |

*Note:* All means and standard deviations were first converted to z-scores before the regression analyses were performed.

^a^For the age group adults were coded as 0 and children/adolescents as 1, therefore a positive value means lower frequency for adults.

^b^For the CHR symptom group perceptive symptoms were coded as 0 and non-perceptive symptoms as 1, therefore a positive value means lower frequency for perceptive symptoms.

*significant at p<0.05, **significant at p<0.01, ***significant at p<0.001

| **eTable 7**. Mixed-effects linear regression for ‘Model2_rmssd’ with age group (children/adolescents vs. adults), CHR symptom group (non-perceptive vs. perceptive symptoms), and their interaction term as predictor | | | | | | |
| --- | --- | --- | --- | --- | --- | --- |
|  | Contrast | SE | z | *p* | 95%CI; lower | 95%CI; upper |
| Age group effect^a^ | 0.191 | 0.194 | 0.981 | 0.327 | -0.190 | 0.571 |
| CHR symptom group effect^b^ | 0.001 | 0.045 | 0.031 | 0.976 | -0.086 | 0.089 |
| Age group x CHR symptom group effect^a^ | 0.363 | 0.091 | 3.997 | 0.000*** | 0.185 | 0.540 |
| Age group effect for perceptive symptoms^a^ | 0.467 | 0.206 | 2.265 | 0.024* | 0.063 | 0.871 |
| Age group effect for non-perceptive symptoms^a^ | 0.104 | 0.195 | 0.533 | 0.594 | -0.279 | 0.487 |

*Note:* All instability indices derived from the mean square rooted successive differences (RMSSD) and their standard deviations were first converted to z-scores before the regression analyses were performed.

^a^For the age group adults were coded as 0 and children/adolescents as 1, therefore a positive value means lower frequency for adults.

^b^For the CHR symptom group perceptive symptoms were coded as 0 and non-perceptive symptoms as 1, therefore a positive value means lower frequency for perceptive symptoms.

*significant at p<0.05, **significant at p<0.01, ***significant at p<0.001

**Results of the sensitivity analyses for the COVID-19 outbreak and global pandemic. Excluding one patient who used the EMA in the week when the lockdown in Switzerland was announced (n=65)**

| **eTable 8**. Mixed-effects linear regression for ‘Model2_mean’ with age group (children/adolescents vs. adults), CHR symptom group (non-perceptive vs. perceptive symptoms), and their interaction term as predictor | | | | | | |
| --- | --- | --- | --- | --- | --- | --- |
|  | Contrast | SE | z | *p* | 95%CI; lower | 95%CI; upper |
| Age group effect^a^ | 0.527 | 0.191 | 2.759 | 0.006** | 0.153 | 0.902 |
| CHR symptom group effect^b^ | 0.011 | 0.037 | 0.297 | 0.766 | -0.062 | 0.084 |
| Age group x CHR symptom group effect^a,b^ | 0.249 | 0.076 | 3.280 | 0.001** | 0.100 | 0.398 |
| Age group effect  for perceptive symptoms^a^ | 0.717 | 0.200 | 3.591 | 0.000*** | 0.326 | 1.108 |
| Age group effect  for non-perceptive symptoms^a^ | 0.468 | 0.192 | 2.438 | 0.015* | 0.092 | 0.844 |
| COVID-19 effect^c^ | 0.098 | 0.188 | 0.521 | 0.602 | -0.271 | 0.468 |

*Note:* All means and standard deviations were first converted to z-scores before the regression analyses were performed.

^a^For the age group adults were coded as 0 and children/adolescents as 1, therefore a positive value means lower frequency for adults.

^b^For the CHR symptom group perceptive symptoms were coded as 0 and non-perceptive symptoms as 1, therefore a positive value means lower frequency for perceptive symptoms.

^c^For the COVID-19 pandemic group after the COVID-19 outbreak was coded as 0 and before the COVID-19 outbreak as 1, therefore a positive value means lower frequency for after COVID-19.

*significant at p<0.05, **significant at p<0.01, ***significant at p<0.001

| **eTable 9**. Mixed-effects linear regression for ‘Model2_rmssd’ with age group (children/adolescents vs. adults), CHR symptom group (non-perceptive vs. perceptive symptoms), and their interaction term as predictor | | | | | | |
| --- | --- | --- | --- | --- | --- | --- |
|  | Contrast | SE | z | *p* | 95%CI; lower | 95%CI; upper |
| Age group effect^a^ | 0.243 | 0.183 | 1.331 | 0.183 | -0.115 | 0.602 |
| CHR symptom group effect^b^ | 0.013 | 0.043 | 0.299 | 0.765 | -0.071 | 0.096 |
| Age group x CHR symptom group effect^a^ | 0.356 | 0.087 | 4.098 | 0.000*** | 0.186 | 0.526 |
| Age group effect for perceptive symptoms^a^ | 0.515 | 0.195 | 2.645 | 0.008** | 0.133 | 0.896 |
| Age group effect for non-perceptive symptoms^a^ | 0.159 | 0.184 | 0.862 | 0.389 | -0.202 | 0.520 |
| COVID-19 effect^c^ | 0.299 | 0.180 | 1.665 | 0.096 | -0.053 | 0.652 |

*Note:* All instability indices derived from the mean square rooted successive differences (RMSSD) and their standard deviations were first converted to z-scores before the regression analyses were performed.

^a^For the age group adults were coded as 0 and children/adolescents as 1, therefore a positive value means lower frequency for adults.

^b^For the CHR symptom group perceptive symptoms were coded as 0 and non-perceptive symptoms as 1, therefore a positive value means lower frequency for perceptive symptoms.

^c^For the COVID-19 pandemic group after the COVID-19 outbreak was coded as 0 and before the COVID-19 outbreak as 1, therefore a positive value means lower frequency for after COVID-19.

*significant at p<0.05, **significant at p<0.01, ***significant at p<0.001

**Results of the sensitivity analyses for age as continuous variable (n=66)**

| **eTable 10**. Mixed-effects linear regression for ‘Model2_mean’ with continuous age, CHR symptom group (non-perceptive vs. perceptive symptoms), and their interaction term as predictor | | | | | | |
| --- | --- | --- | --- | --- | --- | --- |
| **Overall model: χ^2^_(3)_=16.87; p<0.001***** | | | | | | |
|  | Contrast/  Effect | SE | z | *p* | 95%CI; lower | 95%CI; upper |
| Age (continuous) effect | -0.053 | 0.021 | -2.49 | 0.013* | -0.095 | -.0.011 |
| CHR symptom group contrast^a^ | 0.513 | 0.162 | 3.18 | 0.001** | 0.197 | 0.831 |
| Age x CHR symptom group contrast^a^ | 0.027 | 0.008 | 3.27 | 0.001** | 0.011 | 0.044 |
| Age effect for perceptive symptoms | -0.074 | 0.022 | -3.32 | 0.001** | -0.117 | -0.030 |
| Age effect  for non-perceptive symptoms | -0.046 | 0.021 | -2.18 | 0.030* | -0.088 | -0.005 |

*Note:* All means and standard deviations were first converted to z-scores before the regression analyses were performed.

^a^For the CHR symptom group perceptive symptoms were coded as 0 and non-perceptive symptoms as 1, therefore a positive value means lower frequency for perceptive symptoms.

*significant at p<0.05, **significant at p<0.01, ***significant at p<0.001

| **eTable 11**. Mixed-effects linear regression for ‘Model2_rmssd’ with continuous age, CHR symptom group (non-perceptive vs. perceptive symptoms), and their interaction term as predictor | | | | | | |
| --- | --- | --- | --- | --- | --- | --- |
| \| **Overall model: χ^2^_(3)_=23.07; p<0.001***** \| \| --- \| | | | | | | |
|  | Contrast/  Effect | SE | z | *p* | 95%CI; lower | 95%CI; upper |
| Age (continuous) effect | -0.032 | 0.020 | -1.59 | 0.113 | -0.071 | 0.008 |
| CHR symptom group contrast^a^ | 0.806 | 0.182 | 4.41 | 0.000*** | 0.448 | 1.164 |
| Age x CHR symptom group contrast^a^ | 0.043 | 0.009 | 4.53 | 0.000*** | 0.024 | 0.061 |
| Age effect for perceptive symptoms | -0.064 | 0.021 | -3.01 | 0.003** | -0.106 | -0.023 |
| Age effect for non-perceptive symptoms | -0.022 | 0.020 | -1.08 | 0.282 | -0.062 | 0.018 |

*Note:* All instability indices derived from the mean square rooted successive differences (RMSSD) and their standard deviations were first converted to z-scores before the regression analyses were performed.

^a^For the CHR symptom group perceptive symptoms were coded as 0 and non-perceptive symptoms as 1, therefore a positive value means lower frequency for perceptive symptoms.

*significant at p<0.05, **significant at p<0.01, ***significant at p<0.0

**eFigure1:** Contrasts children/adolescents vs. adults for both standardized outcomes (mean and RMSSD) on overall CHR symptoms and symptom group level (non-perceptive vs. perceptive) with 95% confidence interval.


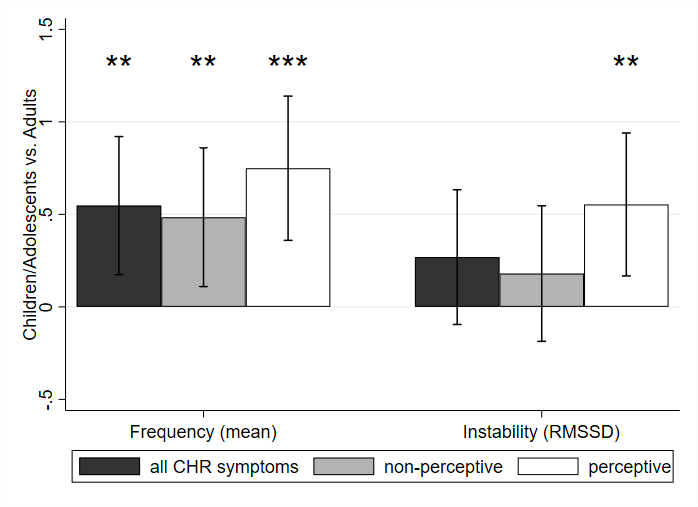


*Note:* *significant at p<0.05, **significant at p<0.01, ***significant at p<0.001; error bars indicate confidence intervals; bar graphs indicate the following effects: age group effect (black), age group effect for non-perceptive symptoms (grey), age group effect for perceptive symptoms (white).
